# Characterization of Dystrophin-Related Syndromes: Carriers, DMD, and BMD

**DOI:** 10.3390/muscles5030060

**Published:** 2026-08-26

**Authors:** Naoufel Chabbi, Corrado Angelini, Irune García, Clara Lépée Aragón, Alicia Aurora Rodriguez

**Affiliations:** 1 Abulkacem Chebbi Medical Center, Tunis 1089, Tunisia; 2 Campus Biomedico Pietro D’Abano, University of Padova, 35128 Padova, Italy; corrado.angelini@unipd.it; 3 Neuro-e-Motion Research Team, Faculty of Health Sciences, Department of Psychology, University of Deusto, 48007 Bilbao, Spain; irune.garciaurquiza@deusto.es (I.G.);

**Keywords:** Duchenne Muscular Dystrophy, Becker Muscular Dystrophy, Pseudometabolic Dystrophinopathic Syndrome, asymptomatic dystrophinopathy, brain dystrophin-related syndrome, X-linked dilated cardiomyopathy (XLDCM), female dystrophin-related syndrome

## Abstract

Primary dystrophin deficiency, caused by X-chromosome mutations within the *DMD* gene, encompasses a continuous clinical spectrum of neurological, muscular, and cardiac disorders known as dystrophinopathies that exhibit profound phenotypic variability driven by specific mutation profiles and epigenetic factors. This comprehensive review analyzes the clinical and molecular characteristics of seven primary classifications: Duchenne muscular dystrophy (DMD), a severe childhood myopathy caused by a complete absence of the protein that leads to loss of ambulation and fatal cardiorespiratory failure in youth; Becker muscular dystrophy (BMD), a milder variant with partial protein deficiency that preserves walking capabilities into adulthood and prolongs life expectancy; pseudometabolic dystrophinopathic syndrome, featuring exercise intolerance, cramps, and recurrent rhabdomyolysis that mimics metabolic diseases; asymptomatic dystrophinopathy, representing the mild end of the spectrum identified incidentally through chronically elevated creatine kinase levels; brain dystrophin-related syndrome, where the disruption of distal isoforms like Dp140 and Dp71 results in neurodevelopmental and neuropsychiatric comorbidities such as ADHD, autism, and intellectual disability; X-linked dilated cardiomyopathy (XLDCM), a cardiac-selective condition causing severe heart failure and arrhythmias while sparing skeletal muscle function; and female dystrophin-related syndrome, distinguishing between familial carriers—who can manifest symptoms due to skewed X-chromosome inactivation—and rare sporadic females who develop an exceptional, severe, Duchenne-like phenotype due to cytogenetic accidents such as Turner syndrome or chromosomal translocations. Ultimately, advancements in molecular testing (NGS and WGS) have significantly optimized diagnostic precision, proving essential for implementing early cardioprotective care, accurate genetic counseling, and the development of future tissue-specific targeted gene therapies. The present study also discusses the psychosocial impact that the disease has on patients.

## 1. Introduction

Primary dystrophin deficiency causes several distinct neurological syndromes, all linked to X-chromosome mutations within the Duchenne muscular dystrophy (*DMD*) gene. A complete absence of the protein results in DMD, a severe, progressive muscular atrophy in children that is typically fatal by the second to third decade due to cardiorespiratory failure, and which affects roughly 1 in 3500 to 5000 male neonates. Partial deficiencies resulting from less frequent allelic variations give less severe dystrophin-related syndromes, including Becker muscular dystrophy, X-linked dilated cardiomyopathy, female dystrophinopathies, isolated intellectual disability, pseudo-metabolic myopathy, and asymptomatic presentations.

Primary dystrophin deficiencies encompass a broad phenotypic continuum driven by specific mutation patterns and isoform disruptions (Table 1).

Significant phenotypic variability has been a recognized feature of dystrophinopathy cohorts since the earliest clinical publications [1]. This variability correlates closely with specific DMD mutations [2], making the underlying mechanisms a broad and dynamic focus of basic and clinical research [3].

Clinical differentiation between DMD and BMD is based on variations in the age of onset, age at loss of ambulation (LOA), and overall life expectancy, with borderline cases classified as intermediate muscular dystrophy (IMD). Intra-familial variability is well-documented, occurring even in monozygotic twins with identical mutations, alongside high phenotypic diversity among Becker muscular dystrophy (BMD) patients [4]. In DMD, variability also extends to the selective involvement of muscle groups and organs, inconsistent central nervous system involvement, and differential sensitivity to glucocorticoids. This clinical diversity is driven by both the mutation spectrum and epigenetic regulatory factors. Historically, the severe DMD phenotype versus the milder BMD phenotype was explained by quantitative dystrophin levels: out-of-frame mutations result in a complete absence of the protein, while in-frame mutations allow for partially functional, truncated dystrophin expression [5].

Rather than distinct entities, dystrophinopathies are widely considered points along a continuous disease spectrum. Reflecting this, the 2022 263rd ENMC International Workshop proposed subclassifying these conditions according to the primarily affected tissue, resulting in categories like skeletal muscle, cardiac, and cognitive dystrophinopathies [6]. This syndromic grouping is already utilized de facto in clinical settings, facilitating clearer genotype–phenotype correlations [3].

Dystrophinopathy-related syndromes exhibit profound phenotypic diversity. Characterizing the distinct clinical features of each syndrome is essential for accurate phenotypic prediction, genetic counseling, proactive complication monitoring, and the implementation of personalized therapeutic strategies.

Although extensive research has individually characterized classic phenotypes such as DMD and BMD, the rapidly expanding clinical spectrum of DMD-related disorders, including brain dystrophin-related syndromes, X-linked dilated cardiomyopathy (XLCM), and the underdiagnosed spectrum of symptomatic female carriers, is frequently presented in a fragmented manner across medical literature. While recent technological breakthroughs in next-generation sequencing (NGS) and whole-genome sequencing (WGS) have revolutionized early detection, a critical gap remains: the lack of a cohesive, integrated conceptual framework that directly bridges specific mutation dynamics and tissue-specific dystrophin isoform disruption with their corresponding clinical manifestations.

While several narrative reviews on dystrophinopathies have been published in recent years, this manuscript uniquely unifies tissue-specific isoform biology, epigenetic modifiers, advanced translational biomarkers, and therapeutic breakthroughs into a cohesive multi-systemic paradigm. Furthermore, it bridges these molecular advances with clinical management frameworks for neglected cohorts, including female carriers and transitional adult populations. It also provides a comprehensive and unified synthesis of dystrophinopathies. Rather than presenting a series of isolated disease descriptions, the primary objective of this manuscript is to elucidate the common molecular thread unifying these entities.

Over the past decade, remarkable advancements have transformed the clinical management of dystrophinopathies. The widespread adoption of NGS and WGS has drastically accelerated molecular diagnostic timelines and refined mutation profiling [3,4]. Early newborn screening initiatives measuring serum creatine kinase levels have gained global momentum [7], while updated multidisciplinary care guidelines have standardized the use of corticosteroids and proactive surveillance, significantly extending patient survival into adulthood [8,9,10].

Despite these groundbreaking achievements, major clinical challenges persist. The rapidly expanding spectrum of non-classic phenotypic presentations, such as brain dystrophin-related syndromes [11,12], isolated X-linked dilated cardiomyopathies [13], and the highly variable spectrum of symptomatic female carriers [14,15], is frequently addressed in a fragmented manner across specialized literature. Furthermore, translating complex genomic variants and tissue-specific isoform disruptions into unified prognostic models remains a critical gap in routine clinical practice [6].

By consolidating these multi-systemic manifestations and diagnostic paradigms into an integrated diagnostic and pathophysiological model, this review seeks to provide clinicians and researchers with a practical, holistic roadmap to refine clinical decision-making, optimize molecular diagnostic workflows, and inform emerging precision medicine strategies for DMD-related disorders.

## 2. Clinical Spectrum of Dystrophin-Related Syndromes

Mutations in the *DMD* gene result in a wide continuum of disorders that extend well beyond classic skeletal muscle pathology. Understanding this phenotypic heterogeneity is essential for early recognition, accurate prognosis, and tailored patient management across different age groups. This section provides a comprehensive overview of the diverse clinical presentations of dystrophinopathies, from classic muscular phenotypes to isolated organ involvement and symptomatic female carriers.

### 2.1. Duchenne Muscular Dystrophy

DMD is a rare disease characterized by the hereditary absence of dystrophin in children. It is listed as OMIM 310200 according to the Online Mendelian Inheritance in the OMIM catalog “https://www.omim.org/” (accessed on 19 May 2026). The condition is fatal within two to three decades and constitutes a great burden for patients, their families, and society.

DMD is the most frequently diagnosed childhood myopathy [16] affecting boys and only exceptionally, girls. Its incidence, ranging from 1/3500 to 1/5000 newborn males, appears to remain relatively stable over time in epidemiological studies, despite progress in genetic counseling and the practice of cascade screening. This stability could be partly explained by the occurrence of de novo cases [17]. While its prevalence is estimated at 2 to 12/100,000 males across various cohorts, its global prevalence is estimated at 4.8/100,000 [18]. These figures likely reflect the balancing effect of preventive genetic counseling on one side and extended survival through improved care on the other [10].

The clinical presentation of patients with DMD varies according to stage of life. At birth, the newborn appears normal despite having very high creatine kinase (CK) levels [7]. However, decreased fetal movement is often retrospectively reported. In the early ambulatory stage from 3 to 5 years old, the diagnosis is typically suggested by a delay in walking, often characterized by toe-walking, hyperlordosis, calf hypertrophy, and difficulty rising from the floor, known as Gowers’ sign.

In the late ambulatory stage, the child’s apparent development often masks the disease’s progression. While clinical stability may appear as a plateau between the ages of five and seven, this period is nonetheless characterized by increasing difficulties in running, climbing stairs, walking, and rising from the ground. In some cases, these motor challenges are accompanied by academic difficulties and intellectual disability. The early non-ambulatory stage begins at about age 10 when the child cannot walk more than 10 m without help [19].

LOA and wheelchair confinement go hand in hand with the onset of scoliosis, contractures, and atrophy, sometimes masked by obesity; at this stage, the (CK) values are usually decreased. The late non-ambulatory stage is characterized by progressive weakness of the upper limbs, from the neck flexors to the distal muscles, alongside marked scoliosis and contractures. Respiratory disorders develop and pose a life-threatening everyday problem, aggravated by heart failure. Constipation, an increased need for care, physical dependence, sadness, withdrawal, and drowsiness are signs of poor quality of life (QOL); on average, death occurs between the ages of 20 and 30 years due to cardiorespiratory failure. Improved care and the generalization of glucocorticosteroids have delayed the average age at death (AaD) [8]. At this stage, the disease poses significant challenges in the management of adult Duchenne patients [9]. A lesser severity, characterized by the loss of ambulation occurring later yet before the age of 16, has led some authors to distinguish an intermediate Duchenne–Becker syndrome [20].

### 2.2. Becker Muscular Dystrophy

BMD (OMIM 300376) is a less severe and rarer dystrophinopathy than DMD; its incidence is estimated at 1 case per 100,000 newborn males and its prevalence at 2 to 9 per 100,000 males [21]. Clinically, BMD has a later onset than Duchenne muscular dystrophy, occurring after age five, and ambulation beyond age 16 can be maintained for a lifetime. Life expectancy (LE) may be prolonged up to the seventh decade [22]. Cardiomyopathy is common, with more than half of the cases presenting as subclinical or dilated cardiomyopathy, altering the prognosis [23]. Intellectual disability affects 7 to 25% of patients, who otherwise maintain an average IQ [24]. Some authors have expanded the clinical definition of BMD to include all non-Duchenne dystrophinopathies, while others have distinguished ‘isolated quadriceps myopathy’, specifically characterized by exaggerated quadriceps atrophy. The disease inheritance is consistent with X-linked transmission, where 100% of daughters are carriers of the mutated X and sons are unaffected.

Clinically, BMD mimics limb–girdle muscular dystrophies (LGMDs) and spinal muscular atrophy (SMA) type 3; their definitive distinction is molecular [25]. Moreover, cardiac monitoring and care are crucial for both Becker patients and their female relatives who are carriers.

BMD cohort studies seek to establish clinical–protein–genomic correlations for a better understanding of the pathogenesis of dystrophinopathies and the factors of variability, with their relative benignity having become an objective of gene therapies in DMD.

### 2.3. Pseudometabolic Dystrophinopathic Syndrome

This rare dystrophinopathic syndrome can manifest in children or adults as a chronic state of exercise intolerance characterized by cramps, myalgias, and stiffness. In some instances, it constitutes an early clinical presentation of BMD, with which it shares the same molecular pathogenesis.

This syndrome can mimic metabolic diseases, such as glycogen storage disorders, fatty acid oxidation disorders, and mitochondrial cytopathies, or other muscular dystrophies, from which biochemical and genetic testing can distinguish it.

A dystrophinopathic nature is suspected in the presence of recurrent episodes of rhabdomyolysis and pigmenturia triggered by intense exercise, fever, cold exposure, or inhaled anesthetics (e.g., desflurane, halothane). These symptoms are often associated with myalgia, muscle weakness, persistently elevated CK levels between episodes, calf hypertrophy, or the familial nature of their occurrences [26].

Rhabdomyolysis, a serious complication of dystrophinopathies, represents acute muscle necrosis characterized by peak CK levels, myoglobinuria, and occasionally malignant hyperthermia. It is life-threatening due to the risks of renal insufficiency, hyperkalemia, and cardiac arrhythmia, necessitating emergency treatment.

### 2.4. Asymptomatic Dystrophinopathy

Rarely, individuals present with persistently elevated and isolated levels of CK discovered incidentally [27]; in such instances, asymptomatic dystrophinopathy is suspected. The incidental finding of high CK levels in a sporadic asymptomatic female raises the possibility of her DMD mutation carrier status. Clinical examination and electromyography (EMG) results are typically unremarkable.

This syndrome represents the mildest end of the dystrophinopathy spectrum and is typically caused by in-frame exonic deletions, generally localized within the central rod domain. Specifically, deletions of exons 51–52 and exon 16 have been reported as asymptomatic DMD deletions [28].

The systematic implementation of whole-genome sequencing (WGS) has facilitated the identification of rare, asymptomatic individuals harboring pathogenic DMD mutations without elevated CK levels. Although these patients require routine cardiac monitoring, detecting such variants during preconception genomic screening poses significant ethical dilemmas regarding risk communication and management [29].

### 2.5. Brain Dystrophin-Related Syndrome

Since his initial description, Duchenne [30] noted cognitive impairment in 6 out of 13 children, speech disorders in two, and one case of epilepsy; consequently, brain involvement is now recognized as a core component of dystrophinopathies. In this multi-systemic disease, muscular dystrophy may occur in isolation or be associated with intellectual disability (ID), which is more severe and frequent in Duchenne muscular dystrophy (up to 50%) [12], than in BMD (7% to 25% of cases) [31]. More recently, pure cerebral phenotypes without muscular involvement have been identified through WGS in children with intellectual disability harboring pathogenic *DMD* mutations [32].

The isoforms Dp427n, Dp427b, and Dp427p are all expressed in the brain, while Dp140 and Dp71 are present in high abundance. Specifically, Dp140 is primarily expressed during fetal development; its promoter is located between introns 44 and 45, with translation initiating at exon 51. In contrast, Dp71 translation begins beyond exon 63.

Genotype–phenotype correlation studies in DMD have demonstrated that specific dystrophin mutations and isoforms are involved in central nervous system (CNS) manifestations. Consequently, neuropsychiatric disorders are most frequently associated with variants in the distal region of the DMD gene (beyond exon 45), which impair the expression of Dp140 and Dp71 [33].

Within the brain, dystrophins are localized in the basal ganglia, specifically the amygdala, as well as in the prefrontal cortex, hippocampus, and cerebellum. They are consistently found in the presynaptic position of neurons, integrated into a dystrophin-associated protein complex (DAPC). Dystrophins play a pivotal role, particularly during the developmental stages [34], fulfilling both structural roles and functions related to neurotransmitter signaling and regulation. Specifically, Dp427 is associated with GABA receptors, while Dp71 is involved in glutamatergic transmission and glial cell function [35].

Neuroimaging studies, including magnetic resonance imaging (MRI) and positron emission tomography (PET), conducted on DMD cohorts have demonstrated reduced brain volume, particularly within the prefrontal gray matter, alongside white matter abnormalities. Furthermore, a decrease in prefrontal cerebral blood flow has also been observed [36]. The localization of these morphological alterations correlates closely with the frontal-like neuropsychological profile characteristic of DMD.

The mechanisms underlying neuropsychological disorders in DMD are notably complex. Beyond the critical role of Dp140 and Dp71 deficiency, several other contributing factors have been proposed: reduced cerebral blood flow, impaired glucose metabolism, and deficits in dystrophin-associated proteins. Furthermore, mutations in genes contiguous to the *DMD* gene may play a role, alongside the significant psychosocial impact of physical disability and social isolation on the patient [37].

Central nervous system involvement has been extensively evaluated across various DMD cohorts. Despite methodological heterogeneity, intelligence quotient (IQ) studies consistently conclude that the average score deviates by one standard deviation from the general population, with verbal IQ being more significantly affected than performance IQ [38].

The intelligence quotient (IQ) of patients with DMD is estimated to be below 70 in 27% of cases. Specifically, an isolated Dp427 deficiency results in an average IQ of 96, whereas a combined Dp427 and Dp140 deficit reduces the average IQ to below 75. Furthermore, the loss of both Dp140 and Dp71 is associated with an average IQ of less than 50 [39]. Distinct neuropsychiatric comorbidities in DMD include autism spectrum disorder (ASD) in 15% of patients, intellectual disability (ID) in 17% to 27%, and attention-deficit/hyperactivity disorder (ADHD) in 32% [40]. Additionally, epilepsy occurs in approximately 5% of Duchenne cases, a prevalence five times higher than the 0.5% to 1.4% observed in the general population [41], further evidencing the role of dystrophin in modulating CNS hyperexcitability.

Beyond global intelligence metrics, brain dystrophin deficiency causes profound perturbations in functional neural network architecture. High-density functional magnetic resonance imaging (fMRI) demonstrates altered cortico-cerebellar and prefrontal connectivity, directly impairing executive functioning, cognitive flexibility, and working memory [12]. Dp71 and Dp140 localization in astrocytic end-feet and post-synaptic densities is vital for clustering GABA receptors and regulating glutamatergic homeostasis; their absence compromises long-term potentiation and synaptic plasticity [35]. Furthermore, central dystrophin loss disrupts circadian clock gene expression within the hypothalamus, predisposing patients to fragmented sleep architecture, obstructive sleep apnea, deficits in social cognition, and emotional dysregulation [11].

In children with DMD, impairments in cognition, attention, concentration, and working memory compromise executive functions such as complex planning. These deficits lead to significant challenges in socio-communicative behavior as well as persistent learning disabilities [34]. A meta-analysis encompassing 4202 DMD patients identified significant emotional dysregulation, including mood disorders, anxiety, and depression in 30% of the cohort. Furthermore, autism spectrum disorder (ASD) was reported in 17% of cases, while ADHD affected 26%. Anxiety and depression were prevalent in 27% and 14–22% of patients, respectively. Finally, learning disabilities and language delays were observed in 44% of the population [11].

Currently, cerebral assessment in DMD relies on standardized tools such as the Wechsler Intelligence Scale; however, research is ongoing to identify more specific tests capable of serving as objective brain biomarkers.

The stable and non-progressive nature of neuropsychological symptoms in brain dystrophin-related syndrome (B.D.R.S.) suggests they are determined during neurodevelopment. Specialized rehabilitative and corrective therapies for mental disorders in Duchenne patients have shown promising outcomes when identified and initiated early, significantly enhancing quality of life [42]. Such interventions remain crucial while awaiting the development of effective restorative therapies targeting cerebral dystrophin expression.

### 2.6. X-Linked Dilated Cardiomyopathy (XLDCM)

In the earliest clinical observations of DMD, dating back to 1836, Conte reported that the eldest of two afflicted brothers suffered from concomitant heart disease late in life [43].

Dystrophinopathies are primarily characterized as skeletal muscle disorders, but also involve the cardiac muscle with variable incidence and severity, ranging from subclinical involvement to dilated cardiomyopathy across the different dystrophinopathy syndromes. Furthermore, due to extended survival through improved management and the refinement of diagnostic tools, the observed prevalence of cardiac impairment has significantly increased.

In 1990, Nigro et al. [44] reported cardiomyopathy in 60–90% of DMD patients and in 60% of BMD patients. Similarly, Muchir et al. [45] identified clinical or subclinical cardiac involvement in 25% of cases by the age of 6, reaching a prevalence of 90% in male dystrophinopathies beyond the age of 18. Regarding genetic carriers, Hermans et al. [46] observed that 8–18% of female carriers presented with dilated cardiomyopathy (DCM).

Cardiac involvement in dystrophinopathies has been correlated with both genetic [47] and epigenic factors [48]. The severity of the cardiopathy appears to be influenced by the patient’s level of physical activity; notably, in Duchenne patients, reduced mobility results in lower cardiac demand, often leaving symptoms latent or subclinical for extended periods. Conversely, in X-linked dilated cardiomyopathy (XLDCM), the preserved skeletal muscle function allows for exertion that rapidly exhausts the selectively affected myocardium, leading to severe, acute heart failure as the primary clinical presentation [49].

Cardiac evaluation in dystrophinopathies has evolved beyond conventional echocardiographic ejection fraction measurement. Cardiac magnetic resonance (CMR) imaging utilizing late gadolinium enhancement (LGE) enables early detection of subepicardial focal fibrosis—typically originating in the inferolateral left ventricular wall—long before systolic dysfunction becomes clinically apparent (Florian et al., 2014) [50]. Myocardial strain imaging via speckle-tracking echocardiography detects subtle regional mechanical dyssynchrony. Early progressive myocardial fibrosis generates arrhythmogenic substrates that predispose patients to ventricular tachycardia and sudden cardiac death, reinforcing the necessity of routine ambulatory Holter monitoring and early initiation of cardioprotective therapy (McNally et al., 2015) [51].

The cardiomyocyte is the site of continuous contractile activity and, similar to skeletal muscle fibers, relies on dystrophin for membrane stability and structural integrity. Although the downstream cellular events exhibit certain differences, cardiac lesions are characterized by myocyte necrosis, limited regenerative capacity, and progressive fibrosis of the left ventricular walls. This process is subsequently followed by ventricular dilation, ultimately leading to myocardial failure and arrhythmia [52].

In 1987, Berko et al. [53] described a large family with several male members suffering from isolated heart failure; the affected adolescents exhibited a severe clinical course, with mortality occurring before the age of 20, whereas female carriers were less severely affected. In this form of dilated cardiomyopathy (DCM), which lacks clinical evidence of myopathy despite elevated CK levels, Towbin [54] identified the involvement of the *DMD* gene through linkage analysis.

Muntoni et al. [55] identified a deletion of exon 1 involving the muscle promoter in a family with XLDCM. Since then, XLDCM has been documented by numerous authors as associated with *DMD* mutations occurring frequently, though not exclusively, at the proximal end of the gene.

The dystrophinopathic nature of dilated cardiomyopathy can be seen in young patients presenting with signs of isolated primary acute heart failure once myositic, viral, or ischemic etiologies have been excluded [56]. Subtle myopathic features may be present, such as elevated CK levels, muscle cramps, or even calf hypertrophy, often alongside a history of familial involvement.

As cardiomyopathy represents the leading cause of death in dystrophinopathies [57], pharmacological interventions, specifically cardioprotective agents and glucocorticoids, which have demonstrated significant efficacy [58,59], should be initiated early through the screening of individuals potentially harboring the DMD mutation. Standard non-specific cardiac drug therapy is typically prescribed, including angiotensin-converting enzyme inhibitors (ACEIs), angiotensin II receptor blockers (ARBs), and beta-blockers (BBs), alongside diuretics and digoxin in cases of manifest heart failure. Furthermore, invasive cardiological interventions may be required for managing arrhythmias, extending in extreme cases to heart transplantation. These strategies remain essential while awaiting efficient dystrophin-restorative therapies, particularly gene-based approaches designed to target the myocardium [60].

### 2.7. Female Dystrophin-Related Syndrome

Two sisters with presumed DMD were reported as early as 1870 by Meryon [1]; however, within the context of this X-linked recessive disease, the condition in females was long considered incidental. Until recently, their role was perceived as being limited to transmitting DMD variants to male offspring, leading to a historical neglect of their clinical study [14]. Currently, modern molecular diagnostic methods have revealed a higher relative frequency of dystrophinopathic females, sparking increased research interest in this subgroup. Expanding our knowledge of the clinical and molecular aspects of female dystrophinopathy [15] should facilitate a deeper understanding of the pathogenic mechanisms of dystrophinopathies in general. Furthermore, this shift is essential for implementing diagnostic and management strategies specifically adapted to female cases, while a standardized classification would promote their inclusion in clinical trials “https://clinicaltrials.gov/” (accessed on 21 May 2026).

Dystrophinopathy in females is characterized by its rarity, as well as the diversity of its phenotypic expressions and underlying genomic mechanisms. This section defines the genotype–phenotype presentations that clinicians may encounter in practice. Clinically, females with dystrophinopathy typically present in two primary ways: most frequently as the mother or female relative of a male proband with DMD or BMD, or, more rarely, as a sporadic female proband presenting with active myopathy.

#### 2.7.1. Familial Dystropinopathic Females

Mothers who transmit a DMD variant are generally presumed to harbor this variant within their genome; however, this is not invariably the case. Depending on the results of the genetic analysis, two distinct statuses may be identified.

Two-thirds of these mothers test positive for their son’s variant and are subsequently identified as carriers. This status may be shared by other female relatives within the family. Furthermore, carrier status may be strongly suspected in the mother, even prior to genomic testing, based on the presence of clinical signs of dystrophinopathy or the existence of another affected relative outside immediate siblings. Although female carriers (heterozygous recessive) are theoretically asymptomatic, CK levels are notably elevated in 50% to 70% of cases [61], and abnormalities on muscle MRI may also be observed [62].

Subtle clinical signs of dystrophinopathy are identified in 10% to 20% of female carriers, who are referred to as manifesting carriers. These clinical features include muscle cramps, myalgia, exercise intolerance, calf hypertrophy, and proximal lower-limb weakness, which may occasionally present asymmetrically. Furthermore, cardiomyopathy develops during life progression in 7.3% to 16.7% of carriers [63]. Notably, dystrophin-negative fibers are observed on muscle biopsies in 26% of asymptomatic (non-manifesting) carriers, even when clinical symptoms are absent [64].

The occurrence of such dystrophinopathic manifestations in female carriers has been attributed to an epigenetic phenomenon known as ‘skewed X-chromosome inactivation’ [65]. In female mammals, the inactivation of one X chromosome typically occurs during the early stages of embryogenesis, forming the Barr body, thereby silencing the expression of its genes. This mechanism ensures that both X chromosomes are not simultaneously active, preventing the redundant synthesis of X-linked proteins.

The selection of the X chromosome to be inactivated is typically a stochastic process, known as random X-chromosome inactivation. However, the preferential inactivation of the X chromosome carrying the wild-type (normal) allele may lead to the clinical manifestation of X-linked pathologies. Consequently, the analysis of the ratio of active-to-inactive X chromosomes has been proposed to evaluate the prognosis of female carriers in dystrophinopathies. It can be calculated using DNA from lymphocytes or muscle fibers; notably, a higher X-chromosome-inactivation (XCI)-skewed ratio correlates with reduced dystrophin production and, subsequently, a more severe clinical phenotype [66].

However, subsequent reports have demonstrated a poor correlation between X-chromosome inactivation (XCI) ratios, dystrophin levels, and mutated transcript abundance, as well as clinical severity in female carriers; it has been suggested that addressing methodological biases might improve this correlation [67]. Moreover, in a multicenter cohort study, this explanation for the onset of dystrophinopathic symptoms in female carriers was questioned, particularly given the observed tissue-specific differences in XCI ratios and their variability with age. Consequently, other regulatory factors are currently being investigated to elucidate the mechanisms underlying these clinical manifestations [68].

Conversely, one-third of mothers are identified as non-carriers. In these instances, the transmitted mutation arose spontaneously (‘de novo’) within the gametes during germ cell proliferation or at the time of fertilization [69]. This status is typically suggested when the proband is a sporadic case, and the mother remains asymptomatic with normal CK levels, unremarkable muscle MRI findings, and no detectable abnormalities on muscle biopsy. Such a status is subsequently confirmed by negative genetic testing.

The recurrence risk for ‘de novo’ mutations is not negligible; the occurrence of the disease in a second child is attributed to the phenomenon of germline mosaicism [70]. This implies that the mother harbors, within her ovaries, a mutated cell line alongside healthy germ cells; this line contains a spontaneously mutated X chromosome, which can lead to multiple affected offspring. The recurrence risk has been reported to vary between 2% and 15%, depending on the study cohort.

The recurrence risk is estimated to be between 14% and 20% in cohorts where haplotype analysis demonstrates the mother-to-child transmission of the risk haplotype. Conversely, this risk decreases to 4.3% in de novo DMD/BMD families where haplotyping has not been performed [71].

To address the risk of late-onset complications in female carriers, international consensus guidelines mandate structured surveillance protocols [15]. Female carriers should undergo baseline clinical neuromuscular assessment, serum CK measurement, and baseline cardiac evaluation (including an ECG and cardiac MRI or a baseline echocardiogram) starting in late adolescence or upon diagnosis, repeated every 3 to 5 years. Prior to pregnancy, carriers require dedicated reproductive genetic counseling, non-invasive prenatal testing (NIPT), or pre-implantation genetic diagnosis (PGD) options. Manifesting carriers presenting with muscle weakness or cardiac involvement should receive tailored physical therapy and early cardioprotective pharmacotherapy identical to affected males.

#### 2.7.2. Sporadic Dystrophinopathic Females

Within this category, exceptional cases of ‘Duchenne-like’ females have been reported, with an incidence estimated at 1 in 50,000,000 female births [72]. Some affected girls carry a *DMD* gene variation on one X chromosome, while the second X chromosome, which should typically provide protection, is neutralized by a cytogenetic accident. Documented cases include Turner syndrome, where one X chromosome carries a DMD mutation and the other is totally or partially deleted [73]; girls presenting with maternal uniparental disomy of the mutated X chromosome [74]; and those possessing a mutation on one X chromosome alongside an autosomal translocation with a breakpoint at Xp21 on the second, which undergoes skewed X-chromosome inactivation (SCXI) [75].

Hence, rare instances of females with two DMD variants have been identified, such as cases of compound heterozygosity involving two distinct DMD mutations [76], as well as anecdotal reports of females with homozygous mutations inherited from both maternal and paternal X chromosomes [77].

Elsewhere, dystrophinopathy may manifest as a sporadic ‘LGMD-type’ female proband. In such cases, a heterogeneous phenotype may be encountered, ranging from isolated hyperCKemia or cramps with exercise intolerance to benign adult myopathy or severe adolescent myopathy. Clinical assessment typically reveals a pathogenic DMD variant, identifying these individuals as isolated female carriers [78].

Therefore, when a female presents with signs of myopathy, especially when associated with cardiopathy, a DMD mutation, although rare, should be considered as a differential diagnosis. Thanks to the increasing accessibility of advanced genetic diagnostic methods, the prevalence of detected females with dystrophinopathy is expected to rise. This will facilitate a more comprehensive understanding of the group’s natural history, the identification of new biomarkers, and the early management of muscular, cardiac, and reproductive health, as well as potential inclusion in clinical trials for novel therapies [15].

## 3. Integrated Pathophysiology: Isoforms, Mutation Types, and Genotype–Phenotype Correlations

Although dystrophin-related syndromes manifest across diverse organ systems, they share a common genetic etiology: pathogenic alterations in the *DMD* gene. The remarkably broad clinical spectrum, ranging from severe, fatal muscular dystrophies to isolated cardiomyopathy or neurodevelopmental deficits, is primarily governed by two main molecular determinants: the specific type and location of the mutation, and the selective disruption of tissue-specific dystrophin isoforms.

**Figure 1:**
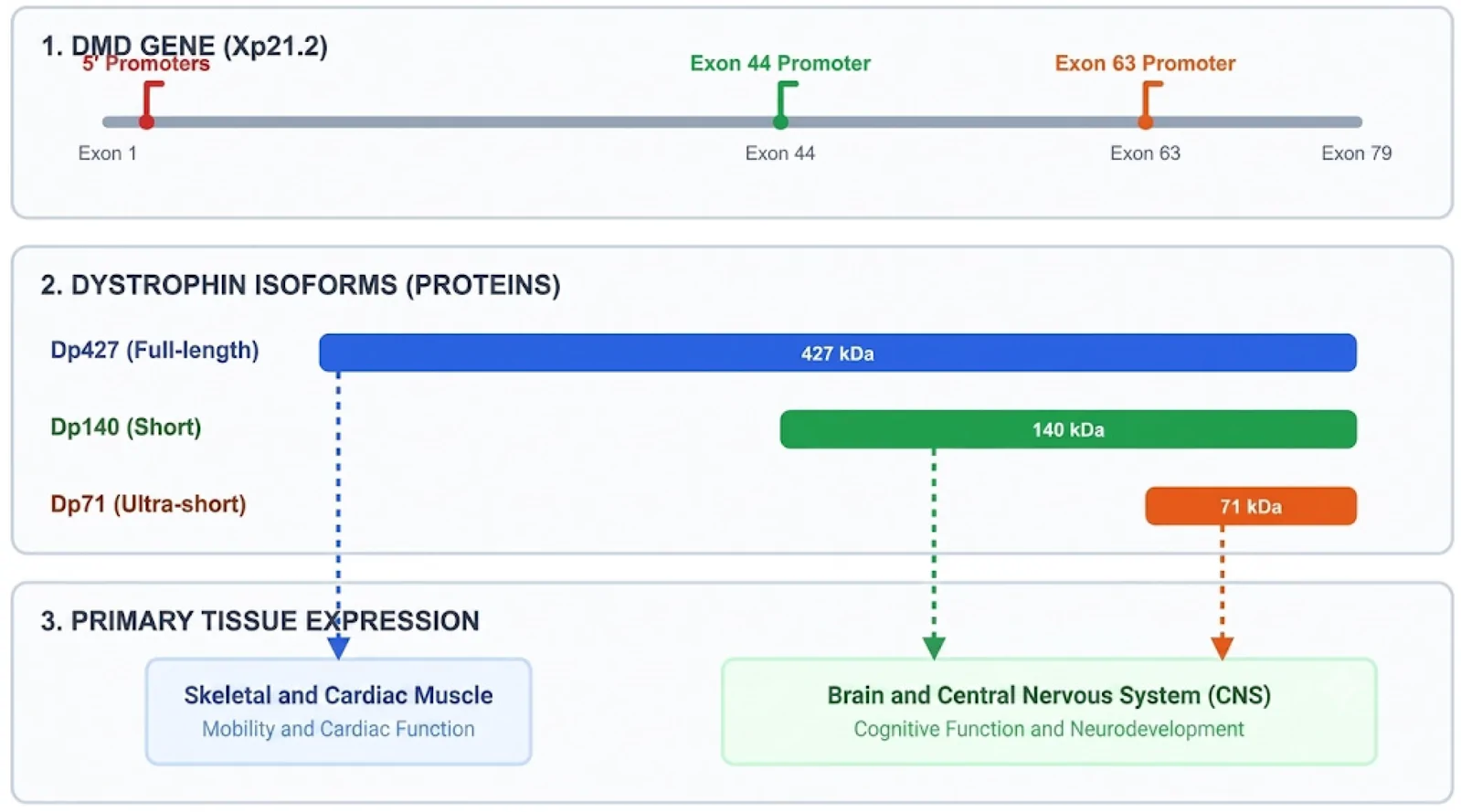
*DMD* gene structure, dystrophin isoforms, and tissue expression.

The DMD gene utilizes multiple internal promoters to generate distinct dystrophin isoforms classified by their molecular weight: full-length isoforms (Dp427m, Dp427c, and Dp427p) and shorter C-terminal isoforms (Dp260, Dp140, Dp116, and Dp71) (Figure 1).

In addition to the full-length Dp427 and the short brain isoforms (Dp140 and Dp71), the *DMD* gene regulates tissue-specific internal promoters that govern Dp260 and Dp116. Dp260 is highly expressed in the retina, where its disruption contributes to altered electroretinogram responses, while Dp116 is primarily expressed in peripheral nerves and Schwann cells, contributing to peripheral nerve myelination and structural integrity [34,79].

**Figure 2:**
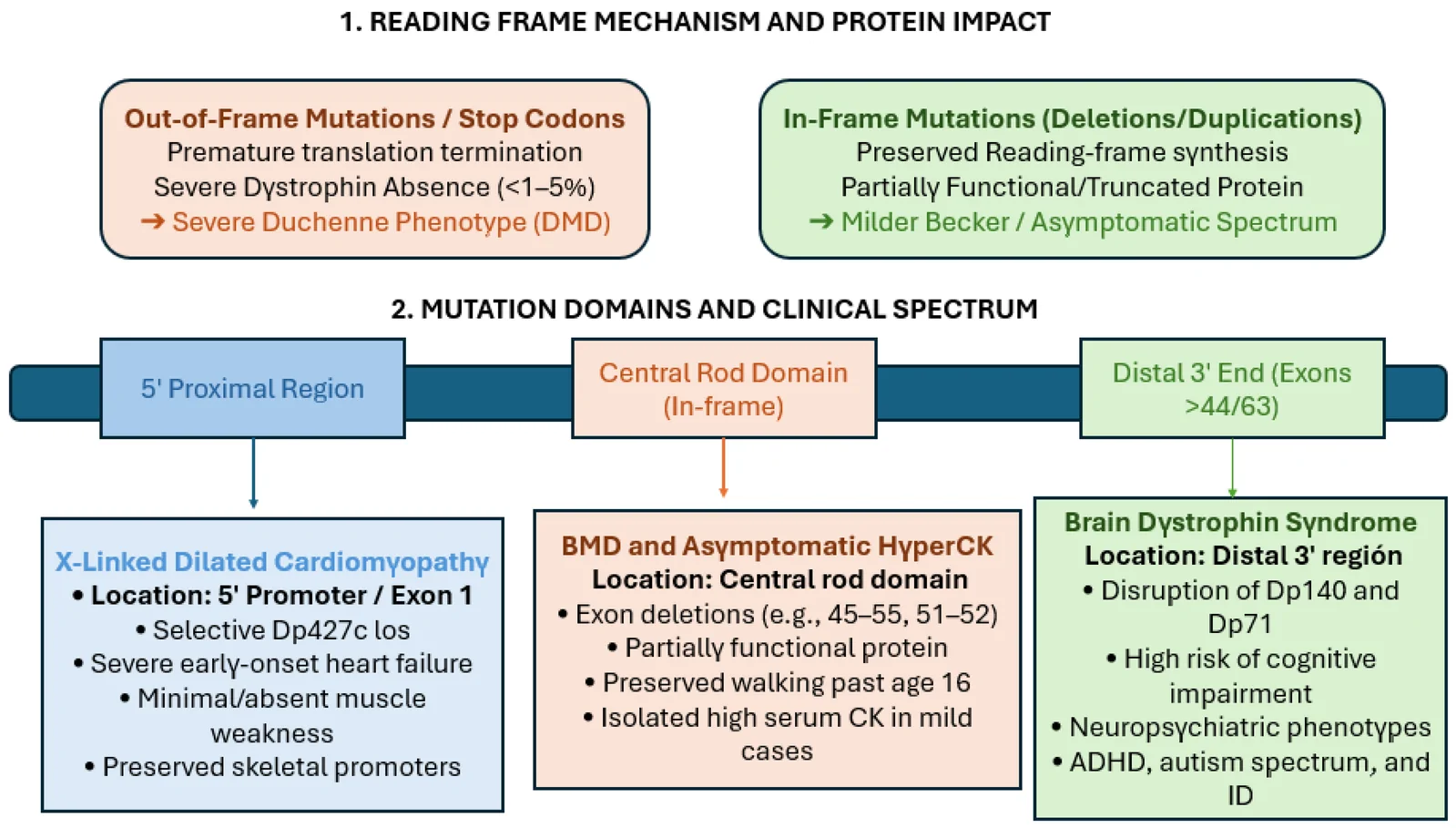
Genotype–phenotype correlations in dystrophinopathies.

Full-length Dp427 is predominantly expressed in skeletal and cardiac muscle, where it forms the dystrophin–glycoprotein complex (DGC) that anchors the internal cytoskeleton to the extracellular matrix, protecting muscle membranes from contraction-induced stress. Consequently, mutations interrupting the open reading frame (out-of-frame mutations) lead to severe Dp427 deficiency, resulting in classical Duchenne muscular dystrophy (DMD). Conversely, mutations that maintain the translational reading frame (in-frame mutations) typically allow the synthesis of a partially functional protein, manifesting as the milder Becker muscular dystrophy (BMD) phenotype (Figure 2) [80,81].

Organ-specific manifestations further depend on which specific promoters and downstream isoforms are compromised. The heart relies almost exclusively on the full-length cardiac isoform Dp427c. In X-linked dilated cardiomyopathy (XLCM), specific mutations near the 5′ end or promoter region selectively impair cardiac transcription while leaving alternative skeletal muscle promoters active, leading to isolated heart failure without significant skeletal muscle weakness. Conversely, central nervous system involvement is driven by shorter isoforms—particularly Dp140 and Dp71—which are heavily expressed in the brain during neurodevelopment and in mature astrocytes and neurons. Mutations occurring toward the 3′ region of the gene, from the distal region to exon 62, disrupt these shorter brain isoforms in addition to full-length Dp427. This cumulative isoform loss explains why patients with distal mutations exhibit a significantly higher incidence of cognitive impairment, neurodevelopmental disorders, and behavioral abnormalities [79].

Finally, female phenotypic heterogeneity highlights the complex interplay between genetic mutation and epigenetic factors. In heterozygous females, phenotypic expression is not solely determined by the underlying mutation type, but by the pattern of X-chromosome inactivation (XCI) during embryonic development. Random skewing of XCI toward the mutant allele leads to variable mosaic expression of functional dystrophin in muscle tissue, ranging from asymptomatic carrier states to severe Duchenne-like phenotypes or isolated late-onset cardiomyopathy. Integrating these mechanisms into a unified framework demonstrates that dystrophinopathies do not represent distinct, unrelated diseases, but rather a continuous clinical spectrum dictated by the spatial, temporal, and structural impact of DMD gene mutations.

### 3.1. Exceptions to the Reading-Frame Rule and Epigenetic Regulation

Although the Monaco reading-frame rule accurately predicts phenotype severity in roughly 90% of cases, substantial clinical exceptions occur [6]. Mild Becker phenotypes resulting from out-of-frame deletions are frequently driven by endogenous exon skipping or alternative splicing events that spontaneously restore the open reading frame. Conversely, severe Duchenne phenotypes presenting with in-frame deletions often stem from disruptions to crucial functional domains, such as the actin-binding N-terminus or the β-dystroglycan-binding C-terminus, or from transcript destabilization via nonsense-mediated mRNA decay (NMD) [82].

### 3.2. Impact of Genetic Modifiers on Disease Progression

Inter-individual phenotypic variability is significantly modulated by secondary genetic loci independent of the primary *DMD* mutation. Latent transforming growth factor-β-binding protein 4 (*LTBP4*) variants modulate TGF-β signaling, with specific protective haplotypes reducing muscle fibrosis and prolonging ambulatory capability [83]. Similarly, osteopontin (*SPP1*) promoter polymorphisms influence inflammatory cascades and glucocorticoid responsiveness [66]. Other established modifiers include *ACTN3* (influencing muscle fiber type composition), *THBS1* (regulating vascular response and ischemia), and *CD40* (modulating systemic cell-mediated immunity), all of which serve as promising targets for adjunctive disease-modifying therapies.

## 4. Molecular Diagnostic Paradigms: From Bench to Precision Therapy

Historically, the diagnostic trajectory for dystrophinopathies relied heavily on invasive approaches, primarily muscle biopsy evaluated via immunohistochemistry and Western blot analysis to demonstrate dystrophin absence or structural alteration [80]. Today, the primary diagnostic paradigm has undergone a transformative shift toward non-invasive molecular approaches, reserving tissue biopsies mainly for unresolved or atypical clinical cases [79,80].

**Figure 3:**
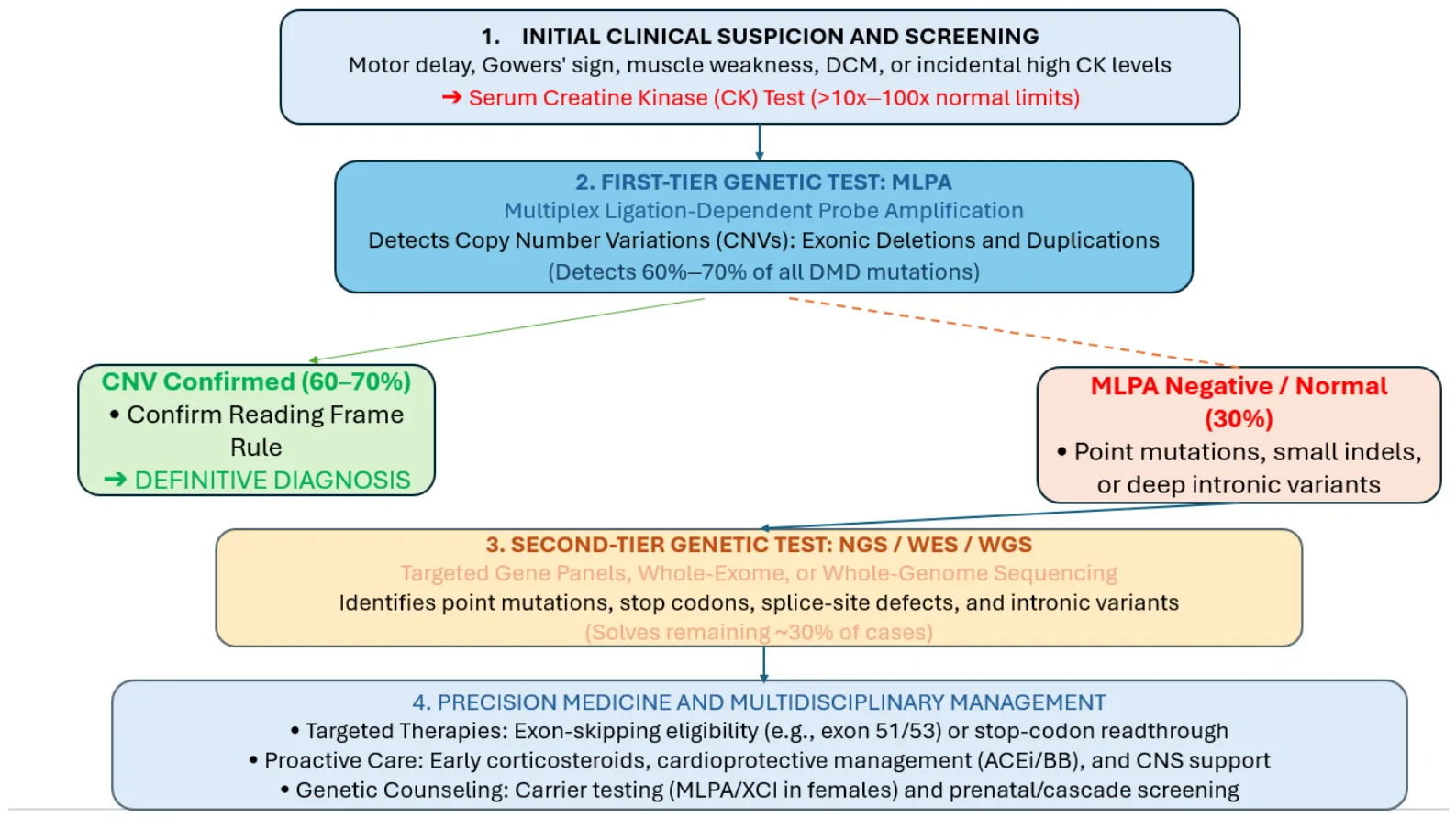
Non-invasive molecular diagnostic workflow and precision medicine paradigm for dystrophinopathies.

Multiplex ligation-dependent probe amplification (MLPA) currently serves as the primary first-tier tool to detect copy number variations, such as large deletions and duplications, which account for approximately 60% to 70% of all DMD mutations [81]. For the remaining 30% of cases caused by small insertions, microdeletions, or point defects, next-generation sequencing (NGS) panels and whole-exome or genome sequencing (WES/WGS) have effectively filled the diagnostic gap (Figure 3) [79]. These high-throughput sequencing pipelines enable rapid, comprehensive screening across the entire *DMD* gene and help identify complex structural rearrangements or deep intronic variants that traditional exon-targeted assays miss [79,81].

Beyond establishing a definitive diagnosis, accurate molecular characterization has become an imperative prerequisite for clinical management and precision therapeutics [80,84]. Early genetic confirmation allows clinicians to initiate proactive corticosteroid regimens, as well as multidisciplinary cardiac and respiratory surveillance, well before substantial muscle loss occurs [80]. Furthermore, definitive molecular profiling dictates patient eligibility for mutation-specific therapies, including antisense oligonucleotide-mediated exon-skipping drugs and nonsense mutation suppression therapies, both of which require precise mapping of deletion boundaries or specific point mutation types [84]. Additionally, rapid molecular diagnosis provides critical support for family genetic counseling, enabling precise carrier detection in female relatives and offering informed reproductive options [80].

Despite these technological breakthroughs, several diagnostic challenges persist in routine clinical practice [79,80]. High-throughput sequencing frequently uncovers novel variants of uncertain significance (VUSs), which often require functional RNA transcript analysis or specialized bioinformatic tools for proper clinical interpretation [79]. Furthermore, diagnosing symptomatic female carriers remains complex, as a heterozygous *DM* mutation detected by MLPA or NGS does not directly predict clinical severity, which is heavily governed by non-random X-chromosome inactivation patterns in target tissues [80]. Finally, significant disparities in financial resources and bioinformatic infrastructure continue to limit universal access to advanced NGS and WGS diagnostic workflows in many global regions [80,81].

### 4.1. Emerging Translational, Fluid, and Digital Biomarkers

To overcome the limitations of invasive muscle biopsies, recent research has prioritized non-invasive biomarkers. Circulating microRNAs (specifically dystromiRs such as miR-1, miR-133a, and miR-206) exhibit high stability in serum and correlate dynamically with muscle degeneration and treatment response [85]. High-sensitivity cardiac troponins (hs-cTnIs) and N-terminal pro-B-type natriuretic peptide (NT-proBNP) serve as early circulating indicators of subclinical cardiomyopathy. Furthermore, serum fibrotic markers (e.g., TGF-β1, collagen VI fragments) and omics-derived proteomic panels offer systemic monitoring of tissue remodeling. In clinical trial settings, digital biomarkers captured via continuous wearable accelerometry (e.g., 95th percentile stride velocity) provide ecological, real-world measurements of motor functional decline [86].

### 4.2. Emerging Therapeutic Strategies

Recent advances in molecular medicine have expanded the therapeutic landscape of dystrophinopathies beyond supportive care towards mutation-specific and disease-modifying strategies. Antisense oligonucleotide-mediated exon skipping has demonstrated the ability to restore the reading frame and promote the production of truncated but functional dystrophin in selected patients, although its efficacy remains mutation-dependent and requires repeated administration [84,87]. Similarly, nonsense read-through therapy offers a treatment option for patients carrying premature stop codons, but its applicability is limited to this specific mutational subgroup and clinical benefits remain modest [87]. Gene replacement using adeno-associated viral vectors encoding microdystrophin has emerged as one of the most promising approaches, while long-term durability, immune responses, and efficient cardiac delivery continue to represent important challenges [87]. Genome-editing technologies based on CRISPR/Cas systems offer the potential for permanent correction of disease-causing variants but require further optimization to improve editing efficiency, delivery, and long-term safety before routine clinical application [88]. In parallel, mutation-independent approaches, including utrophin upregulation, stem-cell-based therapies, and other RNA-based therapeutics, remain under active investigation, although their clinical translation is still limited by biological and technical barriers [87,89]. Overall, future management of dystrophinopathies will likely rely on personalized combination therapies integrating pharmacological, genetic, and regenerative approaches to maximize functional benefit while addressing the heterogeneous molecular mechanisms underlying these disorders [89].

## 5. Psychosocial Impairment of Dystrophin-Related Syndromes

As previously mentioned, dystrophin-related syndromes follow a progressive course and cause significant impairment in most affected individuals. The physical alterations common to all these syndromes are associated with weakness, fatigue, exercise intolerance, pain, and weight-related issues, which in turn lead to a decline in functioning in activities of daily living (ADLs) [90,91]. Therefore, these syndromes have a profound impact on physical and psychosocial health, with loss of mobility serving as one of the primary axes of dysfunction [90].

Likewise, this symptomatology leads to a greater dependence of patients on assistive technology to improve their QOL, as well as on their primary caregivers. Neuromuscular diseases (NMDs) very often cause postural abnormalities and deformities; consequently, most of these individuals will require orthopedic aids [92]. For instance, the majority of patients with DMD require the use of a wheelchair and depend on others for their daily activities. Furthermore, with the onset of chronic respiratory failure, they require assisted mechanical ventilation [93]. Thus, they are placed at higher risk of experiencing poorer social and emotional functioning compared to their healthy peers [94]. In addition, recent research has shown that people with DMD are at greater risk of being victims of bullying and facing difficulties in integrating into the labor market [95].

This vulnerability underscores that the era of defining DMD solely as a pediatric condition is over. However, as these patients survive longer, the transition to adult healthcare systems becomes a critical juncture. An inadequate transition is not merely an administrative inconvenience; it is a direct determinant of health outcomes. Poorly managed transitions are associated with lapses in medical follow-up, lack of adherence to cardiac and respiratory monitoring, increased emergency department utilization, and a palpable sense of abandonment among patients and families. Furthermore, the psychosocial dimensions of this shift, encompassing education, employment, independent living, relationships, and sexual health, are frequently overshadowed by the immediate medical imperatives of the disease [96].

Another issue that arises in the lives of patients and their families is diagnostic delay. The low prevalence of these diseases, coupled with clinical variability, atypical presentations, or a lack of time for a comprehensive examination and medical history collection, contributes to delays in diagnosis [97]. Delayed diagnosis causes feelings of uncertainty in both the patient and the family and constitutes a significant source of stress [98]. Genetic testing has become a critical component of the diagnostic paradigm for neuromuscular disorders; consequently, the identification of novel disease-causing mutations has expanded the understanding of the underlying pathogenic mechanisms of NMDs [99]. It is important to identify ways to reduce these diagnostic delays to provide earlier intervention aimed at improving disease management. A timelier diagnosis would mitigate the impact of the diagnostic odyssey faced by parents and patients, thereby facilitating access to a variety of healthcare interventions [100].

Another challenge faced by these patients and their families is that, since these conditions fall under the category of rare diseases, the available pharmacological treatments to prescribe are classified as orphan drugs. This is because, given their low prevalence, they represent a limited market opportunity for the biopharmaceutical industry, making them less attractive for investment [100]. As a result, therapeutic options for these diseases are often limited; thus, the long-term preservation of health-related quality of life (HRQoL) is frequently the primary focus of medical care [101]. HRQoL refers to the subjective perception, influenced by current health status, of the capacity to perform activities that are important to the individual [102]. It is primarily composed of elements that affect QoL and are related to health, disease, and treatment.

In these types of pathologies, there appears to be a decrease in the HRQoL experienced by the patient due to all the aforementioned factors [42]. It is clear that these conditions have a substantial impact on many or all domains comprising HRQoL, although the impact on different spheres depends on the specific type of disease [102]. In general, NMD-related disabilities have a robust and independent association with all aspects of HRQoL, linked to the inherent symptomatology of these diseases. Specifically, individuals with DMD generally experience a much lower quality of life compared to the general public, a gap that widens with disease progression [103].

However, recent findings indicate that QOL does not necessarily deteriorate with disease progression [42]. Likewise, the caregiver’s role and health have been highlighted as primary predictors of the patient’s quality of life. Therefore, the previous perspective regarding the variables influencing QOL must be modified based on these new findings, and many more variables, such as those previously mentioned, must be considered. Furthermore, considering these aspects that can improve the QOL of these individuals is highly relevant because, in many instances, a treatment is only truly successful if the patient reports feeling better in their daily life. This is why QOL reports are increasingly integrated into protocols evaluating the efficacy of new treatments. Dystrophinopathies are complex and affect multiple areas. By measuring quality of life, the medical team can identify what is impacting the patient most at a specific moment (e.g., fatigue, sleep disturbances, or anxiety regarding loss of mobility).

Therefore, assessing and considering QOL as a relevant variable to analyze the impact of the disease helps direct resources (psychology, physiotherapy, occupational therapy) to where the patient truly needs them most. The need to evaluate quality of life demonstrates that dystrophinopathies are not merely conditions affecting the muscles, but complex diseases that impact the identity, relationships, and holistic well-being of both the individual and their family.

Addressing the complex psychosocial challenges in dystrophinopathies requires structured, multidisciplinary strategies. A comprehensive overview of these domains and proposed interventions is detailed in Table 2.

### Comprehensive Rehabilitation, Physical Therapies, and Assistive Technologies

Long-term management of dystrophin-related syndromes relies heavily on structured rehabilitation programs designed to slow functional decline, mitigate secondary complications, and promote patient autonomy. Current evidence emphasizes submaximal exercise prescriptions, such as low-intensity aerobic training and hydrotherapy, which preserve aerobic capacity and muscle conditioning without exceeding the threshold of contraction-induced sarcolemmal injury or accelerating muscle necrosis [79,92,104]. Conversely, high-resistance strength training and eccentric movements remain strictly contraindicated due to their potential to induce severe rhabdomyolysis and irreversible mechanical degradation of dystrophin-deficient fibers [79,105].

Respiratory rehabilitation plays a critical role in preserving pulmonary function. Proactive protocols incorporating mechanical insufflation–exsufflation (cough-assist devices), nightly non-invasive positive pressure ventilation (NIV), and daily chest physiotherapy significantly reduce pulmonary complications, decrease hospitalizations, and extend overall survival [80,89]. To counteract progressive musculoskeletal degeneration, early intervention with night-time ankle–foot orthoses (AFOs) combined with passive stretching routines helps maintain tendon elasticity, delay joint contractures, and prevent rapid scoliosis progression, often postponing the need for corrective spinal surgery [80,92].

Recent technological advancements have revolutionized functional independence for individuals with dystrophinopathies. Advanced assistive devices, including lightweight custom power wheelchairs, environmental control interfaces, and modern robotic arm orthoses, facilitate mobility and the performance of activities of daily living (ADLs) [80,89]. Furthermore, robotic exoskeletons and wearable powered orthoses represent emerging modalities that offer upper- and lower-limb assistance, enhancing functional reach and reducing metabolic effort during motor tasks [80]. In parallel, digital health solutions, such as telerehabilitation programs and remote wearable sensor monitoring, have expanded access to continuous specialized care, enabling personalized home-based physical therapy routines and real-time tracking of motor function decline [86,89].

## 6. Conclusions

In light of the results, primary dystrophin deficiency caused by *DMD* gene mutations encompasses a highly heterogeneous clinical spectrum, ranging from severe manifestations like Duchenne muscular dystrophy and X-linked dilated cardiomyopathy to milder forms such as Becker muscular dystrophy, pseudometabolic phenotypes, and asymptomatic hyperCKemia. This variability stems from specific mutation profiles and the disruption of distinct isoforms that are vital not only for skeletal and cardiac muscle—where heart involvement represents the leading cause of mortality—but also for the central nervous system, leading to neurodevelopmental and neuropsychiatric comorbidities. Furthermore, dystrophin-related syndromes extend to female carriers and symptomatic females due to mechanisms like skewed X-inactivation or cytogenetic anomalies, while the progressive physical limitations and psychosocial burden highlight the necessity of precise molecular diagnostics, early cardiac monitoring, robust psychosocial support, and structured transitions to adult care.

Collectively, the evidence synthesized throughout this review supports a contemporary view of dystrophinopathies as multisystem disorders in which skeletal muscle degeneration, cardiomyopathy, neurological and cognitive manifestations, and psychosocial challenges represent interconnected consequences of dystrophin deficiency rather than isolated clinical entities [79,80]. This integrated perspective highlights the importance of genotype–phenotype correlations, tissue-specific dystrophin isoform expression, and molecular diagnostics for improving disease characterization, prognostic stratification, genetic counseling, and the identification of patients who may benefit from mutation-specific therapeutic approaches [6,81,83]. Likewise, the implementation of coordinated multidisciplinary care encompassing neuromuscular, cardiac, respiratory, rehabilitation, neuropsychological, and psychosocial management has substantially improved survival and quality of life, reinforcing the need for lifelong, patient-centered care [79,80]. Emerging therapeutic strategies, including exon-skipping, stop-codon read-through, gene replacement, and genome-editing approaches, are progressively reshaping the management of dystrophin-related disorders and moving the field towards increasingly individualized interventions, although their long-term efficacy, accessibility, and equitable implementation remain important challenges [84,87]. Nevertheless, these advances should be interpreted in light of the current limitations of the available evidence, including heterogeneous study designs, variability in outcome measures, limited longitudinal data, and unequal access to specialized diagnostic and therapeutic resources across different healthcare settings. Addressing these gaps through standardized multicenter studies, harmonized outcome measures, broader implementation of precision diagnostics, and equitable access to multidisciplinary care and emerging molecular therapies will be essential to fully realize a unified precision medicine framework for dystrophinopathies, integrating muscular, cardiac, neurological, cognitive, genetic, psychosocial, and therapeutic dimensions into a comprehensive model of patient management.

## Figures and Tables

**Table 1:** Genotype–phenotype correlations and underlying molecular mechanisms in DMD-related dystrophinopathies.

Clinical Syndrome/Phenotype	DMD Mutation Type/Location	Recommended Molecular Testing Strategy	Affected Dystrophin Isoforms	Functional Impact/Protein Expression Pattern
Duchenne Muscular Dystrophy (DMD)	Out-of-frame mutations (deletions/duplications) or premature stop codons (e.g., exons 45–55 or 2–20)	**First-tier:** MLPA to detect copy number variations (deletions/duplications), covering 60–70% of cases. **Second-tier:** NGS panels, WES, or WGS for the remaining 30% to identify point mutations or small indels.	Dp427 (m, c, p) absent; distal short isoforms (Dp140, Dp71) disrupted in 3′ mutations	Near-total absence of functional dystrophin (<1–5%); rapid progressive sarcolemmal instability and muscle degeneration.
Becker Muscular Dystrophy (BMD)	In-frame mutations preserving the reading frame	**First-tier:** MLPA to detect copy number variations (deletions/duplications), covering 60–70% of cases. **Second-tier:** NGS panels, WES, or WGS for the remaining 30% to identify point mutations or small indels.	Dp427 partially preserved, truncated, or internally deleted	Reduced quantity or structurally altered dystrophin; partially functional protein leading to a milder course.
Brain Dystrophin-Related Syndrome	Distal mutations located in the 3′ region (e.g., exons 62–79)	**High-resolution NGS or WGS** focusing on the **distal 3′ region** of the *DMD* gene to identify mutations disrupting Dp140 and Dp71 isoforms.	Short CNS-promoted isoforms (Dp140 and/or Dp71)	Disruption of neuronal, synaptic, and glial functions; manifests as ADHD, ASD, intellectual disability, or emotional dysregulation.
X-Linked Dilated Cardiomyopathy (XLDCM)	5′ region mutations (cardiac promoter/exon 1) or specific structural variants	**Targeted NGS panels for hereditary cardiomyopathies**, with specific analysis of the **cardiac promoter and exon 1** at the proximal 5′ end.	Dp427c severely affected; Dp427m partially preserved in skeletal muscle	Predominantly myocardial deficiency causing cardiac failure/arrhythmias with minimal or absent skeletal weakness.
Asymptomatic HyperCKemia/Myalgias	Specific missense mutations or minor in-frame deletions	**Comprehensive neuromuscular NGS panels** or **WGS** to detect rare in-frame mutations or variants of uncertain significance (VUSs).	Dp427 structurally altered with mild dysfunction	Mild alteration of the dystrophin–glycoprotein complex; elevated serum CK levels with preserved long-term muscle strength.
Female Dystrophinopathy Phenotypes	Pathogenic *DMD* variants modulated by skewed X-chromosome inactivation (XCI) or cytogenetic rearrangements	**MLPA/NGS** for variant detection, supplemented by **X-chromosome inactivation (XCI) ratio analysis** (from lymphocytes or muscle) to correlate with clinical severity.	Mosaic expression of Dp427	Highly variable spectrum, ranging from asymptomatic hyperCKemia to overt Duchenne-like phenotypes or isolated cardiomyopathy.

**Table 2:** Composition of psychosocial challenges and proposed multidisciplinary strategies in dystrophinopathies.

Psychosocial Domain	Core Component/Root Cause	Proposed Strategies and Multidisciplinary Interventions
Emotional Burden and Coping	Chronic stress in caregivers, grief over progressive loss of autonomy, and ambiguous loss	Early and continuous psychological counseling, cognitive behavioral therapy (CBT), and peer support networks
Social and Educational Inclusion	Learning disabilities (linked to Dp140/Dp71 disruption), fatigue, accessibility barriers, and social stigma	Individualized educational plans (IEPs), assistive learning technologies, and school-wide awareness programs
Healthcare Transition	Disruption of care continuity when shifting from pediatric to adult medical systems	Structured transition readiness programs, dedicated adult multidisciplinary clinics, and early transition planning starting in early adolescence
Health-Related Quality of Life (HRQoL)	Severe physical dependency, social isolation, loss of peer engagement, and loss of independence	Routine HRQoL assessment using validated tools (e.g., PedsQL), personal care assistance, home adaptation, and environmental controls

## Data Availability

The data presented in this study are available on request by the corresponding author.
